# Vitamin D status of pediatric epilepsy patients and evaluation of affecting factors

**DOI:** 10.1186/s13052-025-01898-9

**Published:** 2025-02-10

**Authors:** Serap Bilge, Sema Nur Taşkın

**Affiliations:** 1 Department of Pediatric Neurology, Ministry of Health, Children Hospital of Diyarbakır, Diyarbakır, Turkey; 2 Department of Pediatric Rheumatology, Ministry of Health, Children Hospital of Diyarbakır, Diyarbakır, Turkey

**Keywords:** Epilepsy, Pediatric patients, Vitamin D

## Abstract

**Background:**

The use of antiseizure medication in patients with epilepsy is one of the significant risk factors associated with abnormal vitamin D status. We aimed to identify risk factors related to hypovitaminosis D in pediatric patients treated with antiseizure medications.

**Method:**

A cross-sectional retrospective cohort study was conducted on 127 pediatric epilepsy patients who received antiseizure drugs from December 2021 to December 2022. Demographic data, seizure types, diet, physical activity, duration, and types of antiseizure medications were analyzed.

**Results:**

Among the 127 patients in this study, 53% were male, and the mean age was 9,1 ± 4,6 years (range: 2–17). The mean serum 25(OH)D level at baseline in winter/autumn was 24,2 ± 14,2 ng/mL; 47.0% of the patients were 25(OH) D deficient, 23% were 25(OH)D insufficient, and 30% had a vitamin D level within the normal range. The vitamin 25(OH) D level was 27,6 ± 12,2 in the epilepsy group with non-enzyme-induced antiseizure drugs, 21,76 ± 19,7 in the group with enzyme-induced antiseizure drugs, and 13,96 ± 7,9 in the group with combined antiseizure drugs (*p* < 0.001).

**Conclusion:**

The number of antiseizure drugs, treatment with enzyme-induced antiseizure drugs, duration of epilepsy, abnormalities in magnetic resonance imaging, and etiology play important roles in determining the vitamin D level.

## Background

Currently, vitamin D deficiency affects almost everyone worldwide, and it affects not only the musculoskeletal system but also many acute and chronic diseases [1]. Despite its importance, vitamin D is insufficient or deficient in one billion people worldwide [2]. Recent studies have shown that vitamin D deficiency is more common in epileptic patients treated with antiseizure drugs (ASDs), especially drugs that induce cytochrome p450 enzymes [3].

ASDs are categorized according to their ability to induce hepatic cytochrome P450 enzymes: enzyme-induced antiseizure drugs (EIASDs), such as phenytoin, phenobarbital, carbamazepine, eslicarbazepine acetate, primidone, oxcarbazepine, and topiramate > 200 mg/day), and nonenzyme-induced antiseizure drugs (non-EIASDs), such as valproate, lamotrigine, gabapentin, levetiracetam, clobazam, pregabalin, zonisamide, lacosamide, perampanel, topiramate ≤ 200 mg/day, and benzodiazepines [4].

EIASDs can induce cytochrome P450 enzymes, which are responsible for vitamin D catabolism, and consequently convert vitamin D into inactive metabolites [5, 6]. Enzyme-inducing anti-seizure drugs (EIASDs) are known to accelerate vitamin D catabolism through specific biochemical pathways. These drugs include phenobarbital, phenytoin, carbamazepine, and others. The pathways by which they affect vitamin D metabolism are centered on the upregulation of hepatic cytochrome P450 enzymes, particularly CYP3A4 and CYP24A1. Below is an outline of the mechanisms:

1. Induction of Cytochrome P450 Enzymes: EIASDs induce the expression of cytochrome P450 enzymes in the liver by activating nuclear receptors, such as:Pregnane X Receptor (PXR) and Constitutive Androstane Receptor (CAR). These receptors regulate the transcription of genes involved in drug metabolism, including CYP3A4 and CYP24A1, which play roles in vitamin D metabolism.
2. Increased Conversion of Vitamin D to Inactive Metabolites: CYP24A1 enzyme: This enzyme converts 25-hydroxyvitamin D (25(OH)D) and 1,25-dihydroxyvitamin D (1,25(OH)₂D) into inactive forms such as calcitroic acid. Induction of CYP24A1 leads to: Enhanced breakdown of 25(OH)D, lowering its availability for conversion to the active hormone. And to accelerated degradation of 1,25(OH)₂D, reducing the bioactive form’s physiological effects.
3. Impact on Calcium and Phosphorus Homeostasis. Reduced levels of 1,25(OH)₂D impair the intestinal absorption of calcium and phosphorus, contributing to secondary hyperparathyroidism and chronic effects can lead to bone demineralization, osteomalacia, and increased fracture risk.
4. Altered Vitamin D Binding and Bioavailability. Some EIASDs may affect the levels of vitamin D-binding protein, potentially influencing the transport and bioavailability of vitamin D metabolites.

Understanding these pathways highlights the importance of tailored management for individuals on long-term EIASD therapy.

The resulting hypovitaminosis leads to hypocalcemia and an increase in parathyroid hormone levels, increasing bone turnover and increasing susceptibility to osteopenia and bone fractures. Moreover, other major factors related to vitamin D status must be considered, such as insufficient exposure to the sun, geographical factors, skin pigmentation, inadequate diet, and underlying diseases such as malabsorption syndromes, liver disease, and chronic kidney disease [7, 8]. Vitamin D is very important for calcium and bone homeostasis, particularly in children, because childhood and adolescence are the most critical periods for bone development. Moreover, vitamin D not only is essential for bone health but also has important roles in extraskeletal targets, such as the immune system, muscles, and cardiovascular system [1].

Therefore, in this study, we aimed to study and screen for vitamin D deficiency and insufficiency among pediatric epilepsy patients, identify the risk factors associated with vitamin D deficiency/insufficiency and explore the relationship between EIASDs/non-EIASDs and vitamin D status.

## Materials & methods

The medical records of patients aged 2 to 17 years who approached Diyarbakır Children’s Hospital from December 2021 to December 2022 were retrospectively reviewed to detect the following ICD-10 codes: *G40.0* Epilepsy, G40.1 Epilepsy, G41.2 Complex partial epilepsy, G41.8 Other epilepsy, and G41.9 Epilepsy unspecified. Patients with the above ICD codes who adhered to the same antiseizure medication for at least one year with reliable seizure records were included in the study. Demographic and clinical variables, including age, sex, weight, height, body mass index (BMI), age of onset of epilepsy, type of seizure, etiology, predisposing genetic factors, diet, sun exposure, living in the city or urban area, and vitamin D levels, were recorded. The exclusion criteria were changes in the ASD schedule before the study; intake of vitamin D supplementation or medication interfering with vitamin D metabolism six months before the study, use of other drugs in addition to ASDs; history of hypercalcemia, nephrolithiasis, parathyroid disease, or gastric surgery; cerebral palsy; and inability to walk. Clinical and demographic data; vitamin 25(OH) D levels according to the seasons of winter/autumn (December, January, February, September, October, and November)-summer/spring (June, July August, April, and May); Ca, Mg, and PO_4_ levels; onset of epilepsy; number and type of ASDs; duration of medications used; and MRI (normal/abnormal) data were collected through a questionnaire during consultations, follow-up visits, and clinical files. Epilepsy types were considered according to the International League Against Epilepsy (ILAE) classification. Epilepsy was considered refractory when two schedules suitable for maximum tolerable doses of ASDs failed to achieve seizure freedom. Our study was approved by the ethics committee of Health Sciences University Gaziyaşargil Training and Research Hospital, 03–03-2023, with approval number 353.

According to the Endocrine Society Guidelines, vitamin D status is defined as a deficiency when a 25(OH)D level is less than or equal to 20 ng/mL and vitamin D insufficiency when a 25(OH)D level is in the range of 21–29 ng/ml. All vitamin D levels were evaluated via the same laboratory liquid chromatography‒tandem mass spectrometry method in our patients with epilepsy aged 2–17 years from December 2021 to December 2022; serum levels of 25(OH)D were routinely obtained as part of our standard clinical care for patients with epilepsy. In addition, complete blood count; renal function; serum antiepileptic drug levels; and calcium, magnesium, and phosphate levels were assessed [3, 4].

### Statistical analysis

Categorical variables are expressed as numbers and percentages, whereas continuous variables are summarized as the means and standard deviations. To compare categorical variables between the ASDs. The Pearson chi-square test or Fisher’s exact test was used depending on whether the expected value problem arose. The normality of the distribution of continuous variables was confirmed with the Shapiro‒Wilk test. For the comparison of vitamin D levels between two groups (sex, living area, etc.), Student’s t test or the Mann‒Whitney U test was used depending on whether the statistical hypotheses were fulfilled. For comparisons of vitamin D levels between more than two groups (ASD type, epilepsy type, and MRI), one-way ANOVA or the Kruskal‒Wallis test was used depending on whether the statistical hypotheses were fulfilled. For normally distributed data, with respect to the homogeneity of variances, the Tukey or Games and Howell tests were used for multiple comparisons of groups. For nonnormally distributed data, the Bonferroni-adjusted Mann‒Whitney U test was used for multiple comparisons of groups. To evaluate the correlations between measurements, the Pearson correlation coefficient or Spearman rank correlation coefficient was used depending on whether the statistical hypotheses were fulfilled. Linear regression analysis was applied to determine the most effective predictors of vitamin D levels. All analyses were performed via the IBM SPSS Statistics Version 20.0 statistical software package. The statistical level of significance for all tests was considered to be 0.05.

SPSS. Reference (IBM Corp. Released 2011. IBM SPSS Statistics for Windows, Version 20.0. Armonk, NY: IBM Corp).

## Results

Among the 127 patients in this study, 53% were male, and the mean age was 9,1 ± 4,6 years (range: 2–17). The mean serum 25(OH)D level at baseline in winter/autumn was 24,2 ± 14,2 ng/mL; 47,0% of the patients were 25(OH) D deficient, 23% were 25(OH)D insufficient, and 30% registered a vitamin D level within the normal range. The vitamin 25(OH) D level was 27,6 ± 12,2 in the non-EIASD group, 21,76 ± 19,7 in the EIASD group, and 13,96 ± 7,9 in the combined ASD group (*P* < 0,001).

Details of all the clinical and demographic features of the patients are shown in Table 1. The lifestyle habits of all the patients are shown in Table 2. Model parameter estimates of vitamin D according to season are shown in Table 3. A comparison of vitamin D levels according to the season and type of ASD is shown in Table 4. The vitamin D parameters according to the variables are shown in Table 5.

**Table 1:** Clinical and demographic profiles of the patients in the ASD groups

	**Total**	**ASDs type**			**P**
		**Non-EIASDs**	**EIASDs**	**Combined**	
	*N* = 127	*N* = 82	*N* = 24	*N* = 21	
Age*, Mean* ± *SD*	9,1 ± 4,6	9,0 ± 4,6	8,0 ± 5,1	10,8 ± 3,9	0,138
Gender*, n (%)*					
Male	67 (53%)	46 (56%)	9 (38%)	12 (57%)	0,250
Female	60 (47%)	36 (44%)	15 (62%)	9 (43%)	
Weight*, Mean* ± *SD*	38,9 ± 18,4	39,4 ± 19,3	33,2 ± 16,0	43,9 ± 16,1	0,140
Height*, Mean* ± *SD*	132,6 ± 28,8	133,1 ± 28,0	125,4 ± 35,2	139,0 ± 23,1	0,282
BMI*, Mean* ± *SD*	21,1 ± 4,3	21,1 ± 4,5	20,4 ± 3,1	21,9 ± 4,6	0,474
Epilepsy type*, n (%)*					
Focal	57 (45%)	17 (21%)	23 (96%)	17 (81%)	** < 0,001**^**a,b**^
Generalized	58 (46%)	56 (68%)	0 (0%)	2 (10%)	
Combined	2 (2%)	0 (0%)	0 (0%)	2 (10%)	
Unknown	10 (8%)	9 (11%)	1 (4%)	0 (0%)	
Etiology*, n (%)*					
Structural	30 (24%)	11 (14%)	6 (25%)	13 (62%)	** < 0,001**^**b,c**^
Genetic	17 (13%)	8 (10%)	7 (29%)	2 (10%)	
Unknown	76 (60%)	60 (74%)	10 (42%)	6 (29%)	
Other	3 (2%)	2 (2%)	1 (4%)	0 (0%)	
MRI*., n (%)*					
Normal	94 (74%)	70 (85%)	17 (71%)	7 (33%)	** < 0.001**^**b,c**^
Encephalomalacia	13 (10%)	5 (6%)	5 (21%)	3 (14%)	
Leucomalacia	8 (6%)	2 (2%)	1 (4%)	5 (24%)	
Cortical dysplasia	4 (3%)	2 (2%)	0 (0%)	2 (10%)	
Other	8 (6%)	3 (4%)	1 (4%)	4 (19%)	
EEG*., n (%)*					
Normal	2 (2%)	0 (0%)	2 (8%)	0 (0%)	0.059
Epileptic	124 (98%)	82 (100%)	22 (92%)	20 (100%)	
Age of onset*, Mean* ± *SD*	6,1 ± 4.2	6,3 ± 4.2	5,3 ± 4.3	6,4 ± 4,2	0,563
Duration of disease*, Mean* ± *SD*	3,0 ± 2,1	2,7 ± 1,8	2,8 ± 2,2	4,3 ± 2,6	**0,021**^**b,c**^
Number of seizures in last three months*, n (%)*					
0	82 (65%)	56 (68%)	17 (71%)	9 (43%)	0,073
> 1	45 (35%)	26 (32%)	7 (29%)	12 (57%)	
Number of ASDs*, n (%)*					
1	82 (65%)	54 (66%)	22 (92%)	0 (0%)	** < 0,001**^**a,b,c**^
2	24 (19%)	22 (27%)	2 (8%)	16 (76%)	
> 3	21 (17%)	6 (7%)	0 (0%)	5 (24%)	

*SD* Standard deviation

^a^ *p* < 0.05 for NEIAEDs vs. EIAEDs

^b^ *p* < 0.05 for NEIAEDs vs. both

^c^ *p* < 0.05 for EIAEDs vs. both

**Table 2:** Lifestyle habits and laboratory measurements in the ASD groups

	**Total**	**ASDs type**			**P**
		**Non-EIASDs**	**EIASDs**	**Combined**	
	*N* = 127	*N* = 82	*N* = 24	*N* = 21	
Living in a urban*, n (%)*	96 (76%)	63 (77%)	15 (62%)	18 (86%)	0,177
Diet*, n (%)*					
Normal	125 (98%)	82 (100%)	23 (96%)	20 (95%)	0,155
Vegetarian	2 (2%)	0 (0%)	1 (4%)	1 (5%)	
Physical activity*, n (%)*					
< 1 h./week	15 (12%)	9 (11%)	3 (12%)	3 (14)	0,910
> 1 h./week	112 (88%)	73 (89%)	21 (88%)	18 (86%)	
Sun exposure*, n (%)*					
< 1 h./week	13 (10%)	8 (10%)	2 (8%)	3 (14%)	0,831
> 1 h./week	114 (90%)	74 (90%)	22 (92%)	18 (86%)	
Vitamin D level in winter/autumn*, Mean* ± *SD*	24,2 ± 14,2	27,6 ± 12.2	21,7 ± 19.7	13,9 ± 7,9	** < 0,001**^**a,b**^
Vitamin D level in summer/spring*, Mean* ± *SD*	23,2 ± 16,4	26,1 ± 13,2	22,8 ± 29,2	14,2 ± 9,2	** < 0,001**^**a,b**^
Vitamin D level in winter/autumn*, n (%)*					
Deficiency (25(OH)D ≤ 20 ng/mL)	60 (47%)	25 (31%)	17 (%71)	18 (%86)	** < 0,001**^**a,b**^
Insufficiency (25(O.H.)D: 21–29 ng/mL)	29 (23%)	25 (31%)	2 (%8)	2 (%9)	
Normal values (25(OH)D ≥ 30 ng/mL)	38 (30%)	32 (39%)	5 (21%)	1 (5%)	
Vitamin D level in summer/spring*, n (%)*					
Deficiency (25(OH)D ≤ 20 ng/mL)	43 (54%)	19 (36%)	10 (83%)	14 (88%)	** < 0,001**^**a,b**^
Insufficiency (25(O.H.)D: 21–29 ng/mL)	18 (22%)	18 (35%)	0 (0%)	0 (0%)	
Normal values (25(OH)D ≥ 30 ng/mL)	19 (24%)	15 (29%)	2 (17%)	2 (12%)	
Ca*, mg/dL Mean* ± *SD*	10,1 ± 1,3	10,1 ± 1,5	9,9 ± 0,5	10,0 ± 0,6	0,737
Mg*, mg/dL Mean* ± *SD*	2,15 ± 0,58	2,10 ± 0,59	2,2 ± 0.64	2,32 ± 0,48	0,251
PO4*, mg/dL Mean* ± *SD*	2,07 ± 0,47	2,08 ± 0,49	2,02 ± 0,44	2.,0 ± 0,43	0,829

*SD* Standard deviation

^a^ *p* < 0.05 for NEIAEDs vs. EIAEDs

^b^ *p* < 0.05 for NEIAEDs vs. both

^c^ *p* < 0.05 for EIAEDs vs. both

**Table 3:** Model parameter estimates of vitamin D according to season

**Vitamin D Level in Autumn**		
**Variable**	**Estimate**	**95% CI for Estimate**
Number of AED drugs	-6.944	-9.727, -4.161
Living in rural	10.416	5.561, 15,272
NEIAEDs use	8.134	3.835, 12,433
BMI	-0.606	-1.088, -0,124
**Vitamin D Level in Summer**		
**Variable**	**Estimate**	**95% CI for Estimate**
Number of AED drugs	-7.033	-11.038, -3.028
Age	-0.773	-1.477, -0.068
BMI	-1.180	-1.906, -0.453

**Table 4:** Comparison of vitamin D levels according to season and type of ASD

	**N**	**Vitamin D Level**		**P**
		**In Winter/Autumn**	**In Summer/Spring**	
Total	80	23,0 ± 12,9	23,2 ± 16,4	0,901
Non-EIASDs	52	27,4 ± 17,8	26,1 ± 13,2	0,053
EIASDs	12	15,1 ± 8,8	22,8 ± 29,2	0,371
Combined	16	14,6 ± 8,9	14,2 ± 9,2	0,696

**Table 5:** Vitamin D parameters according to the type and number of ASDs

	**Vitamin D level**					
	**N**	**Autumn**	**P**	**N**	**Summer**	**P**
Type of Therapy						
Monotherapy	75	28,1 ± 14,8	**< 0,001**	44	27,2 ± 17,9	**0,002**
Combined therapy	52	18,6 ± 11,2		36	18,3 ± 13,0	
Number of ASDs						
1	76	28,4 ± 15,0	**< 0,001**^**e**^	45	28,1 ± 18,7	**0,001**^**e**^
2	40	19,5 ± 10,7		27	17,9 ± 10,2	
> 3	11	12,6 ± 6,2		8	13,5 ± 10,4	

*e: p* < *0,05 for No. of AEDs is 1 vs. No. of AEDs is 2, and p* < *0,05 for No. of AEDs is 1 vs. No. of AEDs is 3* +

To assess the risk factors associated with the 25(OH)D serum level, six independent variables were found to be statistically significant: number of ASDs (*p* = 0,001), type of ASD (*p* = 0,01), duration of epilepsy (*p* = 0,021), epilepsy type (*p* = 0,001), etiology (*p* = 0,001), and MRI (*p* 0,001).

Due to the high belief that vitamin D is normal/high in sunny seasons, a test for vitamin D levels was performed in 127 patients in winter/autumn, but the same test was performed in only 80 patients in summer/spring. ASD type, epilepsy type, duration of disease, MRI findings, and etiology differed between the groups. Accordingly, the EIASD and combined groups had more patients with focal-type seizures, whereas the non-EIASD group had more patients with generalized-type seizures. Disease duration and the number of attacks in the last three months were greater in the combined group than in the other two groups (non-EIASDs and EIASDs). The percentage of patients with normal MRI findings was greater in the non-EIASD and EIASD groups than in the combined group. In terms of etiology, the rate of patients with structural etiology was greater in the combined group than in the other two groups.

As the number of ASDs used increases, the vitamin D level decreases; however, it is not clear whether this is due to the effect of the drug or the fact that the severity of the disease is greater in patients who use multiple drugs. Therefore, it is necessary to be careful when generalizing and analyzing the results.

## Discussion

Our study highlights the importance of vitamin D deficiency/insufficiency in pediatric patients with epilepsy with ASDs, which should be monitored closely [9, 10]. Our results revealed a high rate of vitamin D deficiency/insufficiency (70%) in winter/autumn and 76% in summer/spring among pediatric epilepsy patients with ASDs, supporting the results of previous reports, and our results were similar to those of the Napakjira Likasitthananon et al. study, which was conducted in Thailand, which revealed that two-thirds of pediatric epilepsy patients had hypovitaminosis D despite living in the tropical zone with abundant exposure to the sun. This finding highlights the effect of ASDs on vitamin D metabolism. Reem Al Khalifah et al. reported the same results in Saudi children with epilepsy who had ASD [3, 11].

Considering the risk factors affecting vitamin D levels, the type of ASD affects vitamin D levels, and EIASDs are accompanied by lower 25(OH) D levels, supporting the results of Inês Antunes Cunha et al. A total of 92 adult patients (44.6% male), with a mean age of 41.0 ± 14.8 years, were included in this study; 56.5% were vitamin D deficient, and 22.8% were vitamin D insufficient, which means that 79.3% of patients had abnormal levels of vitamin D [4].

In agreement with the results of several earlier studies, a greater number of ASDs was associated with lower levels of vitamin D. This result, however, can be explained by the fact that our pediatric patients who had more than one ASD were taking a combination of non-EIASDs and EIASDs and, as a result, were likely to be affected by vitamin D deficiency related to EIASDs, which disagrees with Inês Antunes Cunha et al.’s results, which revealed a greater level of vitamin D in the group that used a greater number of ASDs, which was explained by the fact that most patients who used more than one drug were non-EIASDs in that study [4]. In a study conducted in a tropical area of Malaysia, polytherapy > 1 ASD, age > 12, Indian ethnicity, inadequate sun exposure duration, and female sex were found to be statistically significant risk factors for vitamin D deficiency in pediatric patients with epilepsy [12]. In another study conducted by J H Baek et al., vitamin D levels were lower in patients who had taken anticonvulsants for more than two years than in those who had taken them for less than two years. The same study concluded that those taking oxcarbazepine (EIASDs) had significantly lower vitamin D levels than patients taking valproic acid (non-EIASDs), which was in line with our results to some extent [13]. In our study, the number of ASDs and BMI were found to affect the level of vitamin D in all seasons, whereas age was found to affect the level of vitamin D in summer/spring. These results could be explained by the lower number of patients with vitamin D levels in summer/spring due to the high belief that vitamin D is normal/high in sunny seasons. Living in a rural area affected vitamin D levels in winter/autumn. This situation could be due to the difference in exposure duration to the sun in rural areas in winter/autumn compared with that in urban areas, which may lead to shorter sun exposure. We investigated whether season plays a role in vitamin D levels. Everyone expects to have a relatively high level of vitamin D in summer, but our results revealed that there was insufficient vitamin D in all seasons despite sun exposure for more than one hour a week, which could be due to the use of ASDs. However, only 55% of patients had vitamin D screening tests in the summer/spring. In contrast, all the patients had a test for vitamin D in the winter/autumn seasons for probable vitamin D insufficiency or deficiency due to shorter sun exposure. Therefore, our results could differ if larger samples of vitamin D were investigated in all seasons. In addition, ineffective sun exposure could affect vitamin D levels in summer owing to the use of clothes and sunglasses to avoid strong exposure to ultraviolet sun rays in this very sunny area of our study. In our study, the amount of sunlight received was low in all seasons. In addition, ASDs play a role in vitamin D levels.

In a study conducted in a sunny city in Australia, children with > 2 ASDs or with underlying genetic etiologies were more likely to have vitamin D deficiency. A high percentage of children with long-term ASDs in Australia are at risk of vitamin D deficiency/insufficiency despite living in the subtropics; these results match our results, which were obtained in one of the sunniest cities in Turkey [14].

Investigation of factors that may play a role in vitamin D levels as features of seizures, In our study, patients with focal seizures on EIASDs also had a lower level of vitamin D. We believe that, in addition to the seizure feature itself, enzyme-induced medications may have caused vitamin D deficiency. Abnormal MRI findings and etiology are other important factors that play a major role in indicating vitamin D levels, which could be explained by the use of EIASDs or the use of a greater number of ASDs. A high frequency of seizures in the past three months was not associated with lower vitamin D levels in our study, which conflicts with the findings of Inês Antunes Cunha et al., who conducted a study with adult patients and reported an association between seizure frequency and lower vitamin D levels [4, 5, 13].

Concerning the seizure type and duration of epilepsy, patients who had epileptic seizures for more than four years had lower vitamin D levels. This situation could be explained by the type of ASD used by these patients, mostly EIASDs, and the high number of ASDs. On the other hand, the majority of seizure-free patients used only non-EIASDs, which could account for the higher levels of 25(OH) D observed in these patients. Napakjira Likasitthananon et al. reported that more than two years of ASD use was significantly associated with hypovitaminosis D [3]. This situation could be attributed to different medications being researched simultaneously, and they could influence vitamin D levels through different and several different mechanisms. Moreover, a study by Na Dong et al. revealed that vitamin D levels are lower in epileptic patients before even the start of ASD, which highlights that this disease could also directly affect vitamin D metabolism or that lower vitamin D levels can lead to more seizures, as other neurosteroids, such as vitamin D, are claimed to exert their effects in many ways [15]. Vitamin D plays several roles in the modulation of cell proliferation, differentiation, neurotransmission and the immune response in the central nervous system [16]. Most researchers have focused on the genomic actions of vitamin D, which involves binding of 1,25(OH)D to the nuclear vitamin D receptor and regulating the expression of several proteins in the nervous system, including neurotrophins such as neurotrophin-3, neurotrophin-4, nerve growth factor, and glial cell-derived neurotrophic factor as well as parvalbumin, a calcium-binding protein that inhibits the synthesis of nitric oxide synthetase and prevents seizures [17].

Vitamin D deficiency/insufficiency is common with anticonvulsant therapy, especially EIASDs, which have a particular effect on vitamin D levels. Vitamin D is very important for bone health, and vitamin D deficiency may contribute to many disorders, such as autoimmune, infectious, cancer, degenerative, diabetic, and vascular diseases [18, 19]. Thus, catching a normal level of vitamin D is crucial to avoid most diseases.

### Limitations of the study

Single centers, a limited number of patients, and the unavailability of vitamin D levels in all patients in summer/spring were considered the main limitations of our study.

## Conclusion

This study highlights the high percentage of patients with vitamin D deficiency/insufficiency among pediatric patients with epilepsy. The risk factors for vitamin D deficiency/insufficiency were the number of ASDs, treatment with EIASDs, a long duration of epilepsy and thus a long treatment period, abnormal MRI findings, and etiology, which can necessitate the use of a high number of ASDs. Hence, the vitamin D status of children with ASD should be regularly monitored, and vitamin D supplementation should be considered individually in these patients.

## Data Availability

At the S.B. repository. The datasets used and/or analyzed during the current study are available from the corresponding author upon reasonable request.

## Abbreviations

ASDs: Anti-Seizures Drugs
EIASDs: Enzyme-induced antiseizure drugs
Non-EIASDs: Non-Enzyme-Induced Anti-Seizure Drugs
BMI: Body mass index
ILAE: International League Against Epilepsy
MRI: Magnetic Resonance Imaging
